# Chronic Morphine Treatment Leads to a Global DNA Hypomethylation via Active and Passive Demethylation Mechanisms in mESCs

**DOI:** 10.3390/ijms26157056

**Published:** 2025-07-22

**Authors:** Manu Araolaza, Iraia Muñoa-Hoyos, Itziar Urizar-Arenaza, Irune Calzado, Nerea Subirán

**Affiliations:** 1 Department of Physiology, Faculty of Medicine and Nursery, University of the Basque Country, 48940 Leioa, Spain; manu.araolaza@ehu.eus (M.A.); iraia.munoa@ehu.eus (I.M.-H.);; 2 Bizkaia Health Research Institute, 48903 Barakaldo, Spain

**Keywords:** morphine, development, DNA methylation, mESCs, bioinformatics, passive and active demethylation

## Abstract

Epigenetic regulation, particularly DNA methylation, plays a crucial role in embryonic development by controlling gene expression patterns. The disruption of this regulation by environmental factors can have long-lasting consequences. Opioid drugs, such as morphine, are known to cross the placental barrier and affect the developing central nervous system, yet their precise epigenetic effects during early development remain unclear. This study aimed to elucidate the impact of chronic morphine exposure on the DNA methylation landscape and gene expression in mouse embryonic stem cells (mESCs). mESCs were chronically exposed to morphine (10 μM for 24 h). Genome-wide bisulfite sequencing was performed to identify DNA methylation changes, while RNA sequencing (RNA-Seq) assessed corresponding gene expression alterations. Global levels of 5-methylcytosine (5mC) and 5-hydroxymethylcytosine (5hmC) were quantified using mass spectrometry. Morphine exposure induced global DNA hypomethylation and identified 16,808 differentially methylated genes (DMGs) related to development, cell signalling, metabolism, and transcriptional regulation. Integrative transcriptomic analysis with RNA-Seq data revealed 651 overlapping genes, including alterations in key epigenetic regulators involved on DNA methylation machinery. Specifically, *Tet1* was upregulated with promoter hypomethylation, while *Dnmt1* was downregulated, without changes in promoter methylation after morphine exposiure. Mass spectrometry results confirmed a global decrease in 5mC levels alongside increased 5hmC, indicating the involvement of both passive and active demethylation pathways. These findings demonstrate for the first time that morphine disrupts the epigenetic homeostasis of mESCs by promoting global and gene-specific DNA demethylation, which might be key to the phenotypic changes that occur in adulthood. This work provides novel mechanistic insights into how opioid exposure during early development may lead to persistent epigenetic alterations, with potential long-term implications for neurodevelopment and disease susceptibility.

## 1. Introduction

Nowadays, there is a greater understanding of how environmental factors can heavily impact human health and development. However, the underlying molecular mechanisms involved remain largely unknown. Although epigenetic modifications play a fundamental role in both development and ageing, certain alterations may contribute to onset health issues or diseases, including autoimmune disorders, neurodevelopmental syndromes, cardiovascular diseases, and cancer [1,2]. Prenatal developmental processes are particularly sensitive to environmental exposures and can interfere with normal embryo development, potentially leading to organ dysfunction after birth [3]. Among these environmental factors, morphine is an addictive drug widely used in modern medical practice, whose primary therapeutic value lies in its ability to provide pain relief, or analgesia [4,5]. However, it is well-documented that morphine has numerous adverse physiological side effects on embryonic development [6]. Morphine can readily cross the placental barrier, reaching the developing embryo [7,8] and leading to several problems during the developmental process. Several studies have established that prenatal morphine exposure decreases the weight of major organs during development—including brain, kidneys, and liver in rats [9]—but more critically, impairs normal neural development, causing delays in nervous system maturation [10]. Furthermore, in utero morphine exposure has been associated with altered anxiety-like behaviours, the development of analgesic tolerance, disrupted synaptic plasticity, and structural changes in neurons in the offspring [11,12]. While the primary effects of morphine are mediated through opioid receptors [13], its broader impact on the molecular and epigenetic regulation of embryonic development remains unclear.

During embryogenesis, DNA methylation confers transcriptionally repressive chromatin states at key developmental genes, as it is the mechanism that defines the direction of differentiation at each cell [14,15]. This path is defined during the process of embryogenesis, where DNA methylation patterns guide cellular differentiation and lineage commitment while preserving pluripotency in stem cells [16]. Maintenance of these methylation patterns at differentially methylated regions (DMRs) is critical for normal development, and their loss leads to severe developmental damage process in mammals [17,18,19]. For example, mice embryos lacking the DNA methyltransferases DNMT1 (*Dnmt1-/-*) die, as the development process is disrupted, caused by a deregulation in DNA methylation pattern [20]. Although DNA methylation is generally considered a stable epigenetic modification, it can undergo gradual passive loss during DNA replication across cell generations [21]. Therefore, passive demethylation does not require specific protein machinery, whereas DNA demethylation can also proceed via an active mechanism involving the ten-eleven translocation (TET) protein family [22,23]. DNA demethylation is also a key mechanism in embryogenesis, to ensure the pluripotency of zygotes resulting from the fertilization of gametes, which undergo sequential divisions to drive pluripotent stem cells (PSCs) in the early stages of the embryo [24]. Given the critical role that DNA methylation plays in early development, the present study aims to investigate the epigenetic mechanisms by which chronic morphine exposure alters the developmental potential of embryonic stem cells, with a particular focus on studying DNA methylation and its regulatory machinery in response to morphine.

## 2. Results

### 2.1. Effect of Chronic Morphine Treatment on DNA Methylation in mESCs by Whole Genome Bisulphite Sequencing (WGBS)

**Figure 1:**
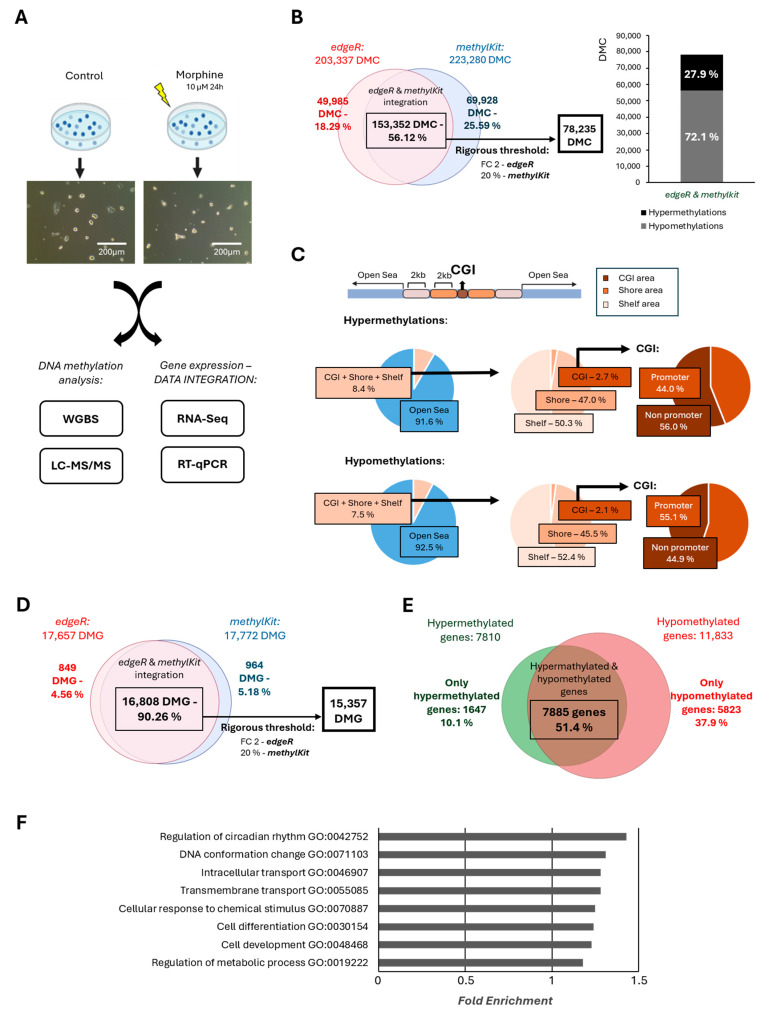
DNA methylation distribution after chronic morphine treatment in mESC by using WGBS. (**A**) Schematic overview illustrating the culture and the exposure to 10 μΜ morphine for 24 h of mESCs (lightning), to determine epigenetic alterations in vitro. Representative images of both treated and untreated mESCs are provided. Scale bar = 200 μm. (**B**) DMC identified through *edgeR* (v.3.32.1) and *methylKit* (v.1.16.1) tools after chronic morphine treatment. Venn diagram shows data integration between both tools. Percentages of hypermethylations and hypomethylations of DMCs form integrative data. (**C**) Pie-chart displaying the CpG feature distribution of DMC across CGIs following chronic morphine exposure, including promoter and non-promoter CGI regions (±1 kb from TSS), shores (<2 kb), shelves (<4 kb), and open sea regions (remaining genome). (**D**) Venn diagram representation of DMGs identified from both *edgeR* and *methylKit* tools. (**E**) Classification of common DMGs based on their methylation status—exclusively hypermethylated, exclusively hypomethylated, or both hypermethylated and hypomethylated genes. (**F**) Functional enrichment analysis highlighting the key biological functions associated with DMGs. Statistical analyses—Fisher’s analyses for *p* > 0.05; n = 4.

First, to investigate the effect of morphine on DNA methylation, we treated mESCs expressing GFP under the Oct4 promoter with 10 μΜ morphine for 24 h, an established in vitro model of chronic morphine exposure [25]. Morphine did not induce any noticeable morphological changes in mESCs (Figure 1A). Next, WGBS was performed to assess genome-wide DNA methylation changes. Using two independent analytical tools—*edgeR* (v.3.32.1) and *methylKit* (v.1.16.1)—203,337 and 223,280 differentially methylated cytosines (DMCs) were identified, respectively, with statistical significance (*p* < 0.05 and FDR < 0.05). Integration of both datasets revealed 153,352 overlapping DMCs, representing 56.12% of the total DMCs identified. (Figure 1B). To increase the biological relevance of the changes, a stricter threshold was applied (fold change ≥ 2 in *edgeR* and ≥ 20% methylation difference in *methylKit*), refining the dataset to 78,235 common DMCs.

Remarkably, the majority of DMCs were hypomethylated, representing 72.1% of the common DMCs, while hypermethylated cytosines accounted for only 27.9%, indicating a widespread reduction in DNA methylation following morphine exposure (Figure 1B). Given the implication of CpG islands (CGIs) in methylation-dependent gene expression regulation [26,27,28], we further analyzed morphine-induced changes in CGIs and flanking features. Although over 90% of DMCs were located in open sea regions, CGIs and their adjacent regions (shores and shelves) were notably enriched in hypermethylated cytosines, suggesting regional specificity despite the overall hypomethylated trend (Figure 1C). Because DNA methylation distribution at promoters also affects gene expression [29], we analyzed DMC distribution in these regions. Promoter-associated DMCs showed a higher frequency of hypermethylation compared to other regions, highlighting that these changes may be relevant to the transcriptional regulation of the associated genes (Supplementary Figure S1).

To gain a comprehensive view of the results, we evaluated how many of these DMCs were located within gene regions, thereby identifying morphine-induced differentially methylated genes (DMGs), as a single gene can contain multiple cytosine residues that may be altered. According to our data, *edgeR* identified 17,657 DMGs, while *methylKit* identified 17,772 DMGs. To ensure the reliability of our results, we focused exclusively on the 16,808 DMGs consistently identified by both tools, representing a 90.26% overlap (Figure 1D). Afterward, we applied more stringent thresholds to further enhance the relevance of the data: a fold change ≥ 2 in *edgeR* and ≥ 20% methylation difference in *methylKit*. As a result, the dataset was further refined to 15,357 DMGs (Figure 1D). Consistent with genome-wide patterns, gene-level analysis revealed that chronic morphine treatment predominantly induced hypomethylation within genes. Most affected genes (51.4%) exhibited both hypermethylation and hypomethylation. However, in 37.9% of cases hypomethylations were exclusively observed in genes related to basic cellular processes such as signalling transduction, apoptosis, metabolism, as well as developmental processes. In contrast, only 10.1% of genes were exclusively hypermethylated, purely involved in primary metabolic processes (Figure 1E, Supplementary Figure S2). To gain further insight into the biological functions in which morphine was involved, Gene Ontology (GO) analysis was conducted. Functional enrichment analysis revealed that morphine-sensitive genes were involved mainly in the regulation of the circadian rhythm, DNA conformational changes, cell differentiation and development and metabolic processes (Figure 1F).

**Figure 2:**
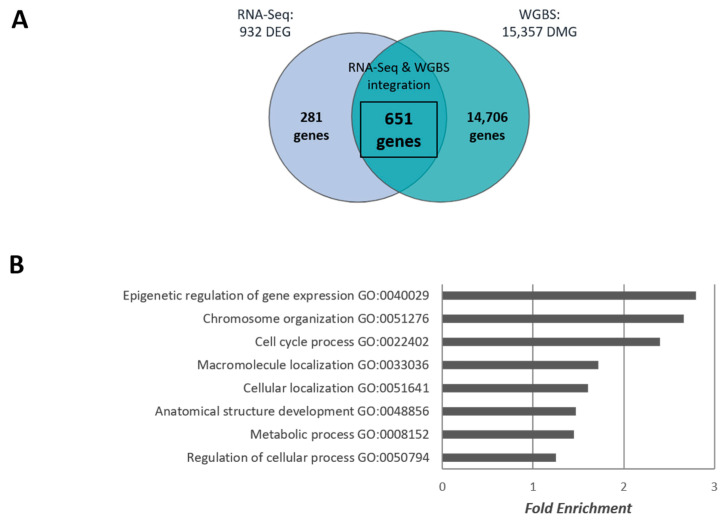
Morphine-sensitive genes from integrative analyses of RNA-Seq (n = 4) and WGBS (n = 4) data. (**A**) Venn diagram illustration of the overlap among DMGs from WGBS data, and RNA-Seq identified DEGs after chronic morphine treatment. (**B**) GO analysis identifying the principal biological functions, conducted using Fisher’s method for *p* < 0.05.

Aiming to understand the relevance of methylome changes in transcriptomic deregulation after chronic morphine treatment, an integrative analysis of WGBS and RNA-Seq data was performed. Specifically, we compared the genes that showed changes in methylation status after morphine exposure (DMG) with those that exhibited changes in expression following morphine treatment (DEG). This integrative approach allowed us to assess whether alterations in DNA methylation were associated with transcriptional regulation, thereby providing additional insights into how morphine exposure may influence gene regulation in mESCs. For that purpose, we used our previously published RNA-Seq data (GEO Store: GSE151234), in which mESCs were exposed to morphine under the same experimental conditions [30]. Briefly, chronic morphine exposure for 24 h resulted in 932 DEGs (Figure 2A), mainly associated with nuclear and cell division, DNA repair, chromosome organization, gene expression, metabolism, and signalling process [30]. A cross-analysis using a Venn diagram identified 651 genes common to both DMGs and DEGs datasets, indicating a strong overlap between methylation changes and transcriptional regulation (Figure 2A). Functional enrichment of these overlapping genes further highlighted involvement in epigenetic regulation, chromosome organization, and cell cycle process, among others (Figure 2B). Specifically, 23 genes associated with epigenetic regulation and gene expression were identified, including core genes belonging to DNA methylation machinery (Table 1). Remarkably, DNA (cytosine-5)-methyltransferase 3-like (*Dnmt3l*) as well as methylcytosine dioxygenases (*Tet1*) were identified as morphine-sensitive genes at transcriptomic and methylome levels, indicating that morphine can potentially self-regulate DNA methylation levels.

### 2.2. Effect of Chronic Morphine Treatment on DNA Methylation Machinery

**Figure 3:**
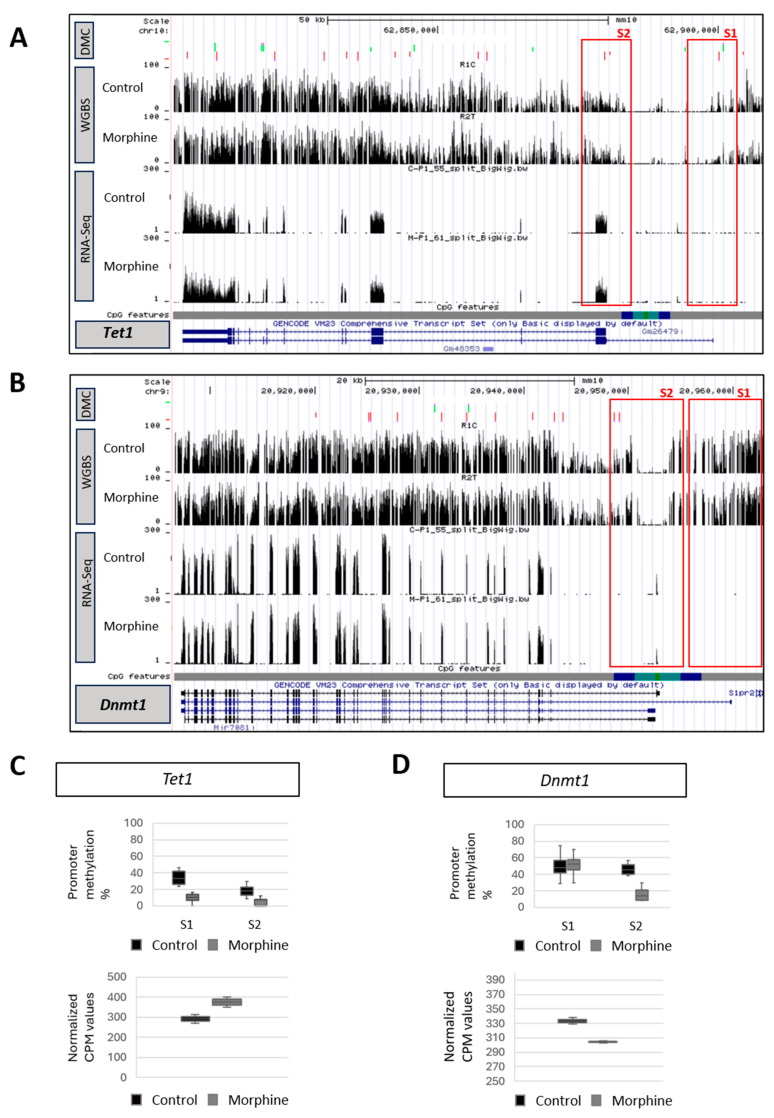

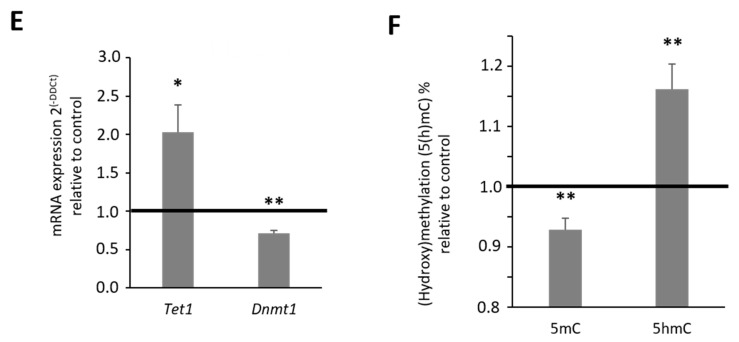
Effect of chronic morphine treatment on DNA methylation and demethylation machinery. RNA-Seq and WGBS track for (**A**) DNA demethylating enzyme *Tet1* gene, and (**B**) DNA methyltransferase *Dnmt1* gene. CpG features a track with CGIs (green), shores (light blue), shelfs (dark blue) and open sea regions (grey). Red boxes highlight areas of enrichment and altered gene expression at promoter sites. In the DMC row, the light green bars represent hypermethylations, and the red bars, instead, hypomethylations. Box and whisker plot showing the percentage of methylation at promoters and CPM values for (**C**) *Tet1* gene, and (**D**) *Dnmt1* gene after chronic morphine treatment. n = 4. (**E**) RT-qPCR analysis for the validation of *Tet1* and *Dnmt1* gene expression. *Gapdh* and *Pcx* served as housekeeping controls and relative expression was calculated using 2ddCT method normalized to untreated controls. n = 5. (**F**) DNA methylation and hydroxymethylation levels after chronic morphine treatment by LC-MS/MS. Percentage of 5mC and 5hmC levels normalized with respect to control. n = 10. Statistical significance was assessed by Student’s *t*-test, denoting different levels: * *p* < 0.05 and ** *p* < 0.01.

WGBS and RNA-Seq analyses confirmed that morphine was able to regulate key components of the DNA methylation machinery in mESCs (Figure 3, Supplementary Figures S2 and S3). The levels and patterns of DNA methylation are dynamically regulated by both DNA methyltransferases (DNMT1, DNMT3A, and DNMT3B) and demethylating proteins, including the ten-eleven translocation (TET) family of dioxygenases (TET1, TET2, and TET3) [31]. Genome browser tracks obtained from UCSC genome browser revealed an effect of chronic morphine treatment not only on *Tet1* and *Dnmt3l* but also on *Dnmt1* gene expression (Figure 3 Supplementary Figure S4A). Regarding demethylating enzymes, morphine led to a decreased DNA methylation level at *Tet1* gene body and specifically promoter region, correlating with an increase in *Tet1* transcript levels (Figure 3A,C). WGBS also proved that morphine altered the DNA methylation pattern on *Tet3* at different regions, including promoter areas, which did not influence its gene expression. Finally, no significant changes in methylation or expression were observed for *Tet2* (Supplementary Figure S3).

Similarly, morphine induced an upregulation of *Dmt3l* gene expression, correlating with a downregulation of DNA methylation at promoter (S3), which controls the majority of transcripts that are expressing (Supplementary Figure S4A,D). While DNMT3L protein is part of the DNA methylation machinery, it is not directly involved in catalytic activity [32,33]. Therefore, we next evaluated the impact of morphine on DNA methyltransferases. Contrary with the results obtained for TET proteins, morphine decreased DNA methylation levels of *Dnmt1* along the whole gene, including the S2 promoter region, which is responsible for expressing 3 out of 4 *Dnmt1* transcripts and corresponds to a CGI region. However, this alteration was not consistent with its reduced gene expression (Figure 3B,D). In contrast to other DNA methyltransferases, morphine mainly induced hypermethylation along the whole *Dnmt3a* gene, which was not associated with changes on gene expression and no changes were reported on the *Dnmt3b* gene after chronic morphine treatment (Supplementary Figure S4).

The observed transcriptomic reduction in the expression of *Dnmt1* maintenance methylase, as well as the significant increase in the expression of *Tet1* demethylase were validated by RT-qPCR, confirming the veracity of the RNA-Seq results (Figure 3E). Finally, to evaluate the impact of *Dnmt1* and *Tet1* gene expression alterations on the whole DNA methylation pattern, global DNA methylation as well as hydroxymethylation levels were measured by mass spectrometry (LC-MS/MS) after chronic morphine treatment. Consistent with previous gene expression results, chronic morphine treatment led to an overall decrease in DNA methylation and a corresponding increase in hydroxymethylation in mESC (Figure 3F). Precisely, morphine induced a 5% reduction in 5mC levels, meanwhile the overall level of hydroxymethylation (5hmC) underwent a significant increase of 15%. These results were in full agreement with those observed with the WGBS technique, where chronic morphine treatment caused a global decrease in DNA methylation. Chronic morphine treatment, therefore, leads to a decrease in cellular methylation levels, generating a global hypomethylated genomic state in the mESC.

## 3. Discussion

Morphine is a well-documented teratogen known to disrupt normal embryonic development, particularly affecting the formation of the neural tube, frontal cortex, and spinal cord, ultimately resulting in delayed nervous system maturation [10]. Although morphine is an addictive substance able to cross the placental barrier and reach the embryo [7,8], the molecular mechanisms through which morphine affects neurogenesis and broader physiological aspects of embryonic development remain incompletely understood. Due to their capacity for indefinite self-renewal and pluripotency, embryonic stem cells are widely employed as in vitro models to study the impact of environmental stimuli on developmental biology [34,35,36]. Our findings prove that DNA methylation represents an important epigenetic mechanism, key to understand how morphine affects early embryonic development, as DNA methylation largely determines the direction of cell differentiation and the proper cellular function [14,15]. Similarly to other addictive substances such as cocaine [37,38] and cannabinoids [39], we demonstrate that chronic exposure to morphine leads to global DNA demethylation in mESCs, as revealed by WGBS analysis. Together with a previously described global downregulation of H3K27me3 in response to chronic morphine treatment [30], the DNA methylation reduction may contribute to the inactivation of a repressive chromatin environment and the promotion of active chromatin regions in mESCs. This effect may be mediated through the activation of opioid receptors, which are present in mESC [40,41]. The morphine-induced epigenetic landscape appears to maintain a pluripotency state of the mESC and avoid cell differentiation as global hypomethylation is a hallmark of pluripotent cells and is essential for preserving their undifferentiated state in proper embryonic development [42,43,44]. This mechanism might also contribute to developmental delays observed in the nervous system [10]. Importantly, morphine affects the DNA methylation distribution of key genes involved in cell differentiation and development, DNA conformation or metabolic processes, beyond the regulation of their gene expression, hence the role of DNA methylation is more complex and nuanced than has been thought.

DNA methylation might mediate the genomic response to morphine through genes involved in the epigenetic regulation of gene expression, chromosome organization, cell cycle or metabolic process among others. Specifically, upregulation of *Tet1*, a key DNA demethylase enzyme, may explain the global hypomethylation induced by morphine [22,23]. Consistent with previously reported epigenetic marks such as H3K27me3 [30], we propose that morphine may have a self-regulation mechanism that modifies *Tet1* gene expression through promoter demethylation, thereby regulating its own expression through positive feedback. In addition, morphine may regulate the expression of *Tet1* through specific transcription factors, such as OCT4 and c-Myc, which have binding sites in its promoter [45,46] and, as previously reported, are upregulated in response to morphine exposure [47]. It is well established that DNA demethylation is a key mechanism in embryogenesis for maintaining pluripotency [48] and this process occurs through an active demethylation by TET enzymes, which leads to the oxidation of 5mC to 5hmC [22,23]. Chronic morphine treatment can induce DNA hypomethylation through this active mechanism, which is also aligned with a significant rise in global DNA hydroxymethylation levels.

In addition to active demethylation, passive mechanisms may also play an important role in overall morphine-induced demethylation [49,50,51]. Our results prove an upregulation of *Dnmt3l* gene expression in response to morphine treatment, which is consistent with a downregulation of DNA methylation levels at gene promoter. DNMT3L is required for germ line DNA methylation, although it is inactive as a DNA methyltransferase per se [32,33]. Previous studies have shown that DNMT3L physically associates with the active de novo DNA methyltransferases, DNMT3A and DNMT3B, and stimulates their catalytic activity [32,33]. However, RNA-Seq data confirmed that morphine decreases *Dnmt1* gene expression, while *Dnmt3a* and *Dnmt3b* remain unchanged. Since DNMT1 primarily maintains the DNA methylation pattern, and DNMT3A/3B methyltransferases are responsible for de novo methylation [20], morphine may compromise the methylation maintenance in mESCs, without significantly altering de novo methylation. Although the sensitivity of DNA methylases to morphine is evident [52,53], it does not alter the promoter methylation status of these genes, implying that additional epigenetic mechanisms or transcription factors will play a crucial role in understanding how morphine regulates these epigenetic enzymes. In neuronal cells, an increase in DNMT1 gene expression via the CREB signalling pathway has been previously reported [54]. This pathway might also link chronic opioid exposure to DNA methylation dynamics, as morphine is known to modulate CREB activity [55]. Reduced *Dnmt1* gene expression may negatively affect the methylation maintenance, leading to passive demethylation rather than initiating a DNA methylation mediated genomic response. Overall, our findings confirm that morphine induces DNA demethylation through both active and passive mechanisms, leading to a further reduction in the already hypomethylated state typical of mESCs [23,56,57,58,59]. Considering the alteration of the DNA methylation pattern, coupled with inhibition of the PRC2 repressive machinery that leads to a global alteration in H3K27me3 chromatin organization [30], morphine promotes an aberrant transcriptome in mESCs. Such epigenetic instability may contribute significantly to abnormal embryo development and lifelong health outcomes [60,61,62].

## 4. Materials and Methods

### 4.1. Cell Culture and Treatment

mESCs (Oct4-GFP cell line) (PCEMM08, PrimCells, San Diego, CA, USA) were maintained under feeder-free conditions on culture dishes coated with 0.1% gelatine (Sigma, St. Louis, MO, USA). The cells were grown in Knock Out Serum DMEM (Gibco, Waltham, MA, USA) supplemented with 15% KSR (Gibco), 1% sodium pyruvate (Sigma), 1% non-essential amino acids (Sigma), 1% penicillin–streptomycin (Sigma), 1% l-Glutamine (Sigma), and 0.07% β-mercaptoethanol (Sigma). Pluripotency was sustained using the LIF+2i condition, consisting of 1000 U/mL leukemia inhibitory factor (LIF) (Sigma), 10 mM PD0325901 (Stemgent, Cambridge, MA, USA), and 30 mM CHIR99021 (StemCell, Vancouver, BC, Canada). To maintain optimal culture conditions, cells were passed every 48 h using trypsin TrypLE Express Enzyme (1×, Thermo Fisher, Waltham, MA, USA), ensuring no attachment for longer than two days. GFP expression from the Oct4-GFP construct was used as an intrinsic marker to monitor pluripotency and prevent spontaneous differentiation. For chronic morphine exposure, mESCs were incubated in their respective culture medium supplemented with 0.9% (*p*/*v*) NaCl for control condition and 10 μM morphine (Alcaliber, Madrid, Spain) for 24 h for treatment condition. Following treatment, cells were collected for downstream analyses.

### 4.2. Cell Lysates, DNA Extraction and Quality Measurement

Genomic DNA was isolated from both control and morphine-exposed mESCs using a DNA lysis Buffer containing 100 mM Tris-HCl, 5 mM EDTA, 200 mM NaCl and 0.2% SDS, supplemented with Proteinase K (AM2546, Thermo Fisher Scientific, Waltham, MA, USA) at a concentration of 100 mg/mL. Samples were incubated overnight under gentle agitation. The following day, 5 μL RNAse (R5125, Sigma) at 10 mg/mL were added to each sample and incubated for 1 h at 37 °C. DNA extraction was performed using the conventional phenol-chloroform/isoamyl alcohol extraction method, employing phenol (P4557, Sigma), chloroform (CL01981000, Scharlau, Sentmenat, Spain), and isoamyl alcohol (BP1150, Fisher BioReagents, Waltham, MA, USA). After the extraction, DNA concentration and purity were determined by measuring the 260/280 absorbance ratio using a Nanodrop ND-1000 Spectrophotometer (Thermo Fisher Scientific).

### 4.3. DNA Methylation Analysis by WGBS

Purified DNA obtained from 4 biological replicates (4 control and 4 treatments) was polled into two technical replicates for each condition and sonicated to generate 300 bp fragments (Soniprep 150). Once denatured, the entire following experimental procedure was carried out in the CRG (Centre for Genomic Regulation, Ciutat Vella, Spain) service. Those fragments were subjected to a DNA bisulfite treatment with EZ DNA Methylation-Lightning Kit, for later analysis. DNA fragments were processed for library construction using a KAPA Library Preparation Kit in combination with xGen^TM^ Methyl UDI-UMI Adapters. The library quality was confirmed using Agilent 2100 Bioanalyzer DNA 7500 assay 0. Following library preparation, 4× multiple sequencing was performed in paired end option on an Illumina NovaSeq 6000 S4 platform (San Diego, CA, USA), with each of the two biological replicates per sample yielding a minimum of 50,000 reads.

### 4.4. Bioinformatics Analyses of WGBS’s Data, and WGBS and RNA-Seq Base Data Integrative Analyses

The quality of the FASTQ files generated from the WGBS data was evaluated using the FastQC High Throughput Sequence QC Report (v.0.11.6) [63], revealing a quality score exceeding 30. Library fragment adapters were cut using Trim Galore! (v.0.6.2) [64], and consequently, the reads obtained were concatenated with Cat (v.8.22) [65]. Alignment of WGBS reads to the UCSC mm10 reference genome was carried out with Bismarck (v.0.22.1) [66,67,68]. The resulting binary alignment files were subsequently sorted and indexed using SAMtools (v.14.0) [69], to enable a more efficient data retrieval. Spearman correlation analysis verified the reproducibility of each sample type. For the identification of the DMC, two different statistical tools were chosen: *edgeR* (v.3.32.1) [70,71] and *methylKit* (v.1.16.1) [72]. The PCA (principal component analysis) in each of the tools confirmed the similarity between replicates and the differences between samples (morphine vs. control). After data normalization, different thresholds for each of the tools were stablished; minimum methylation-percentage difference of 25% (*methylKit*) [72], and minimum fold-change difference of 2 (*edgeR*) [70,71]. Differentially methylated cytosines were considered with a *p* value of ≤0.05 (WGBS-GEO storage: GSE292082). The used RNA-Seq data was previously published by our research group and was generated under the same experimental procedure, analyzing four biological replicates in each condition (RNA-Seq-GEO storage: GSE151234) [30]. Integration of WGBS and RNA-Seq datasets was performed using Venny tools (v.2.1.0) [73], and The Gene Ontology Resource from the GO Consortium (v.16.1.0) (https://geneontology.org/ (accessed on 26 March 2021)) was conducted to identify the biological functions [74,75]. The UCSC genome browser was performed for methylation and gene expression landscape visualization [76].

### 4.5. LC-MS/MS. Mass Spectrometry-Based Quantification of DNA

The isolated DNA underwent enzymatic digestion using DNA Degradase Plus (E2020, Zymo Research, Irvine, CA, USA). The entire following experimental procedure was carried out in SGIker (Servicio Central de Análisis (UPV/EHU), Gasteiz, Spain) service. From each sample 10 μL aliquots containing 50 ng of digested DNA were analyzed using a reversed-phase UPLC system equipped with an Eclipse C18 column (2.1 mm × 50 mm, 1.8 μm particle size, Agilent). The column was equilibrated and eluted at a flow rate of 100 μL/min using a mixture of water/methanol/formic acid, in 95:5:0.1 ratio, (*v*/*v*/*v*). The column effluent was introduced into an electrospray ionization (ESI) source (Agilent Jet Stream) connected to a triple quadrupole mass spectrometer (Agilent 6460/6400 QQQ, Santa Clara, CA, USA). The instrument was operated in positive ion multiple reaction monitoring (MRM) mode under previously optimized conditions. The ionization source parameters were set as follows: capillary voltage at 3.5 kV, nebulizer gas at 40 psi, drying gas flow at 10 L/min, drying gas temperature at 350 °C, and sheath gas temperature at 375 °C with a flow of 11 L/min. The collision energies were optimized as 15 eV for 5mC, 20 eV for 5hmC, and 12 eV for C. Specific ion transitions for the target fragments were measured and recorded as follows: MH^+^→(5mC *m*/*z* 242.1→126.1, 5hmC *m*/*z* 258.1→142.1, and C *m*/*z* 228.1→112.1). The retention times were approximately 3.14 min for 5mC, 4.02 min for 5hmC, and 1.85 min for C. To calculate the percentage of 5mC and 5hmC in each experimental sample, the areas of the obtained MRM peaks were used as a reference and divided by the total amount of cytosines (5mC + 5hmC + C), representing the total cytosine pool.

### 4.6. Real-Time PCR (RT-qPCR)

Total RNA from mESCs was isolated using Nucleozol reagent (Macherey-Nagel, Düren, Germany) in accordance with the manufacturer’s protocol. RNA concentration was measured based on absorbance at 260 nm, while purity was evaluated via the 260/280 nm absorbance ratio. Complementary DNA (cDNA) was synthesized from isolated RNA samples using the iScript cDNA synthesis Kit (Invitrogen, Waltham, MA, USA). RT-qPCR analysis was conducted with the iTaq Universal SYBR Green SuperMix (Applied Biosystems, Foster City, CA, USA) on a PerkinElmer CFX96 Real Time Detection System (BioRad, Hercules, CA, USA). The thermal cycling parameters consisted of an initial denaturation of 39 cycles at 95 °C for 10 min, a hybridization of 20 s at 95 °C, and an annealing and extension of 1 min at 59 °C. All reactions were conducted in triplicate and repeated across a minimum of three independent biological replicates. The level of gene expression was quantified using the ddCT method. Regarding the normalization, two stable housekeeping genes were used, glyceraldehyde 3-phosphate dehydrogenase (*Gapdh*) and pyruvate carboxylase (*Pcx*). The primer sequences used are detailed in Supplementary Table S1.

## 5. Conclusions

Our results provide insights into how transcriptional changes induced by morphine may be mediated by DNA methylation in mESCs. Morphine disrupts selective target genes related to epigenetic regulation, chromosome organization, cell cycle, and, particularly, to cell development and differentiation through alterations in the DNA methylation pattern. Using both active and passive mechanisms that involve the action of demethylases and DNA methyltransferases, chronic morphine treatment causes in vitro global hypomethylation that can maintain the pluripotency state of mESCs. These morphine-induced DNA methylation changes might be key to the phenotypic changes that occur in adulthood, including changes in behaviour, dopamine signalling pathways, synaptic plasticity, and neuronal structures [11,77,78,79], which have a strong potential to persist over time [10,80,81]. Further experiments are required to understand the mechanisms underlying morphine-induced heritable effects, which will be crucial for establishing the foundations of cellular memory in response to external stimuli during embryo development.

## Figures and Tables

**Table 1:** Morphine-sensitive genes related to biological function “Epigenetic regulation of gene expression (GO: 0040029)” that have been identified by integrating RNA-Seq and WGBS databases.

Gene	Full Gene Name
*Glmn*	Glomulin
*Zfp445*	Zinc finger protein 445
*Suz12*	Polycomb protein Suz12
*Smarcad1*	SWI_SNF-related matrix-associated actin-dependent regulator of chromatin subfamily A containing DEAD_H box 1
*Tex15*	Testis-expressed protein 15
*Cbx5*	Chromobox protein homologue 5
*Dnmt3l*	DNA (cytosine-5)-methyltransferase 3-like
*Smchd1*	Structural maintenance of chromosomes flexible hinge domain-containing protein 1
*Zfp869*	Zinc finger protein 869
*Dicer1*	Endoribonuclease Dicer
*Atad2*	ATPase family AAA domain-containing protein 2
*Smarca5*	SWI_SNF-related matrix-associated actin-dependent regulator of chromatin subfamily A member 5
*Tet1*	Methylcytosine dioxygenase TET1
*Klf2*	Krueppel-like factor 2
*Trip12*	E3 ubiquitin-protein ligase TRIP12
*Sirt1*	NAD-dependent protein deacetylase sirtuin-1
*Kdm5a*	Lysine-specific demethylase 5A
*Mettl4*	N(6)-adenine-specific methyltransferase METTL4
*Cbx3*	Chromobox protein homologue 3
*Wbp2*	WW domain-binding protein 2
*Rif1*	Telomere-associated protein RIF1
*Myc*	Myc proto-oncogene protein
*Hells*	Lymphocyte-specific helicase

## Data Availability

Authors consent to the availability of data and materials. The raw data has been deposited in NCBI Sequence Read Archive (SRA) through the Gene Expression Omnibus. WGBS (GEO storage: GSE292082) and RNA-Seq (GEO storage: GSE151234).

## Supplementary Materials

The following supporting information can be downloaded at: https://www.mdpi.com/article/10.3390/ijms26157056/s1.

## Abbreviations

The following abbreviations are used in this manuscript:

5hmC	Hydroxymethylcytosine
5mC	Methylcytosine
cDNA	Complementary DNA
CGI	CpG Island
CPM	Counts Per Million
CT	Cycle quantification value
ddCT	2(-Delta Delta C(T)) method
DEG	Differentially Expressed Gene
DMC	Differentially Methylated Cytosine
DMG	Differentially Methylated Gene
DMR	Differentially Methylated Region
DNA	Deoxyribonucleic acid
DNMT1/3A/3B/3L	DNA methyltransferase 1/3A/3B/3L
ESC	Embryonic Stem Cell
FDR	False Discovery Rate
GAPDH	Glyceraldehyde-3 phosphate dehydrogenase
GFP	Green Fluorescent Protein
GO	Gene Ontology
H3K27me3	Trimethylation of lysine 27 on histone 3 protein subunit
ICR	Imprinting Control Region
KSR	KnockOut Serum Replacement
LC-MS/MS	Liquid Chromatography with tandem mass spectrometry
LIF	Leukemia Inhibitor Factor
mESC	mouse Embryonic Stem Cell
mRNA	messenger RNA
MS/MS	mass spectrometry
OCT4 (POU5F1)	Octamer-binding transcription factor 4 (POU Class 5 Homeobox 1)
PCA	Principal Component Analysis
PCR	Polymerase Chain Reaction
PCX	Pyruvate carboxylase
PE	Paired End
PSC	Pluripotent Stem Cells
RNA	Ribonucleic acid
RNA-Seq	RNA-Sequencing
RT-qPCR	Real Time Quantitative Polymerase Chain Reaction
TET1/2/3	Ten eleven translocation 1/2/3
TSS	Transcription start site
UCSC	University of California Santa Cruz
WGBS	Whole Genome Bisulfite Sequencing

## References

[1] de Gonzalo-Calvo D., Iglesias-Gutiérrez E., Llorente-Cortés V. (2017). Epigenetic Biomarkers and Cardiovascular Disease: Circulating MicroRNAs. Rev. Española Cardiol. (Engl. Ed.).

[2] Moosavi A., Motevalizadeh Ardekani A. (2016). Role of Epigenetics in Biology and Human Diseases. Iran. Biomed. J..

[3] Ko E.B., Hwang K.A., Choi K.C. (2019). Prenatal Toxicity of the Environmental Pollutants on Neuronal and Cardiac Development Derived from Embryonic Stem Cells. Reprod. Toxicol..

[4] Matthes H.W., Maldonado R., Simonin F., Valverde O., Slowe S., Kitchen I., Befort K., Dierich A., Le Meur M., Dollé P. (1996). Loss of Morphine-Induced Analgesia, Reward Effect and Withdrawal Symptoms in Mice Lacking the Mu-Opioid-Receptor Gene. Nature.

[5] Manglik A., Kruse A.C., Kobilka T.S., Thian F.S., Mathiesen J.M., Sunahara R.K., Pardo L., Weis W.I., Kobilka B.K., Granier S. (2012). Crystal Structure of the μ-Opioid Receptor Bound to a Morphinan Antagonist. Nature.

[6] Nakatani T. (2017). Opioid Therapy and Management of Side Effects Associated with Opioids. Gan Kagaku Ryoho.

[7] Kazemi M., Sahraei H., Azarnia M., Dehghani L., Bahadoran H., Tekieh E. (2011). The Effect of Morphine Consumption on Plasma Corticosteron Concentration and Placenta Development in Pregnant Rats. Iran. J. Reprod. Med..

[8] Levitt P. (1998). Prenatal Effects of Drugs of Abuse on Brain Development. Drug Alcohol. Depend..

[9] Eriksson P.S., Rönnbäck L. (1989). Effects of Prenatal Morphine Treatment of Rats on Mortality, Bodyweight and Analgesic Response in the Offspring. Drug Alcohol. Depend..

[10] Niknam N.A., Azarnia M., Bahadoran H., Kazemi M., Tekieh E., Ranjbaran M., Sahraei H. (2013). Evaluating the Effects of Oral Morphine on Embryonic Development of Cerebellum in Wistar Rats. Basic Clin. Neurosci..

[11] Byrnes J.J., Babb J.A., Scanlan V.F., Byrnes E.M. (2011). Adolescent Opioid Exposure in Female Rats: Transgenerational Effects on Morphine Analgesia and Anxiety-like Behavior in Adult Offspring. Behav. Brain Res..

[12] Gapp K., Jawaid A., Sarkies P., Bohacek J., Pelczar P., Prados J., Farinelli L., Miska E., Mansuy I.M. (2014). Implication of Sperm RNAs in Transgenerational Inheritance of the Effects of Early Trauma in Mice. Nat. Neurosci..

[13] Subirán N., Casis L., Irazusta J. (2011). Regulation of Male Fertility by the Opioid System. Mol. Med..

[14] Correa L.O., Jordan M.S., Carty S.A. (2020). DNA Methylation in T-Cell Development and Differentiation. Crit. Rev. Immunol..

[15] Smith Z.D., Meissner A. (2013). DNA Methylation: Roles in Mammalian Development. Nat. Rev. Genet..

[16] Toyooka Y. (2021). Pluripotent Stem Cells in the Research for Extraembryonic Cell Differentiation. Dev. Growth Differ..

[17] Howell C.Y., Steptoe A.L., Miller M.W., Chaillet J.R. (1998). Cis-Acting Signal for Inheritance of Imprinted DNA Methylation Patterns in the Preimplantation Mouse Embryo. Mol. Cell. Biol..

[18] Howell C.Y., Bestor T.H., Ding F., Latham K.E., Mertineit C., Trasler J.M., Chaillet J.R. (2001). Genomic Imprinting Disrupted by a Maternal Effect Mutation in the Dnmt1 Gene. Cell.

[19] McGraw S., Oakes C.C., Martel J., Cirio M.C., de Zeeuw P., Mak W., Plass C., Bartolomei M.S., Chaillet J.R., Trasler J.M. (2013). Loss of DNMT1o Disrupts Imprinted X Chromosome Inactivation and Accentuates Placental Defects in Females. PLoS Genet..

[20] Li E., Bestor T.H., Jaenisch R. (1992). Targeted Mutation of the DNA Methyltransferase Gene Results in Embryonic Lethality. Cell.

[21] Inoue A., Shen L., Dai Q., He C., Zhang Y. (2011). Generation and Replication-Dependent Dilution of 5fC and 5caC during Mouse Preimplantation Development. Cell Res..

[22] Zhao H., Chen T. (2013). Tet Family of 5-Methylcytosine Dioxygenases in Mammalian Development. J. Hum. Genet..

[23] Wu H., Zhang Y. (2014). Reversing DNA Methylation: Mechanisms, Genomics, and Biological Functions. Cell.

[24] Bagci H., Fisher A.G. (2013). Dna Demethylation in Pluripotency and Reprogramming: The Role of Tet Proteins and Cell Division. Cell Stem Cell.

[25] Yang H., Sun J., Chen H., Wang F., Li Y., Wang H., Qu T. (2019). Mesenchymal Stem Cells from Bone Marrow Attenuated the Chronic Morphine-Induced CAMP Accumulation In Vitro. Neurosci. Lett..

[26] Cain J.A., Montibus B., Oakey R.J. (2022). Intragenic CpG Islands and Their Impact on Gene Regulation. Front. Cell Dev. Biol..

[27] Deaton A.M., Bird A. (2011). CpG Islands and the Regulation of Transcription. Genes Dev..

[28] Illingworth R.S., Bird A.P. (2009). CpG Islands—“A Rough Guide”. FEBS Lett..

[29] Weber M., Hellmann I., Stadler M.B., Ramos L., Pääbo S., Rebhan M., Schübeler D. (2007). Distribution, Silencing Potential and Evolutionary Impact of Promoter DNA Methylation in the Human Genome. Nat. Genet..

[30] Muñoa-Hoyos I., Halsall J.A., Araolaza M., Ward C., Garcia I., Urizar-Arenaza I., Gianzo M., Garcia P., Turner B., Subirán N. (2020). Morphine Leads to Global Genome Changes in H3K27me3 Levels via a Polycomb Repressive Complex 2 (PRC2) Self-Regulatory Mechanism in MESCs. Clin. Epigenetics.

[31] Zeng Y., Chen T. (2019). DNA Methylation Reprogramming during Mammalian Development. Genes.

[32] Kareta M.S., Botello Z.M., Ennis J.J., Chou C., Chédin F. (2006). Reconstitution and Mechanism of the Stimulation of de Novo Methylation by Human DNMT3L. J. Biol. Chem..

[33] Guenatri M., Duffié R., Iranzo J., Fauque P., Bourc’his D. (2013). Plasticity in Dnmt3L-Dependent and -Independent Modes of de Novo Methylation in the Developing Mouse Embryo. Development.

[34] Gepstein L. (2002). Derivation and Potential Applications of Human Embryonic Stem Cells. Circ. Res..

[35] Dvash T., Benvenisty N. (2004). Human Embryonic Stem Cells as a Model for Early Human Development. Best Pract. Res. Clin. Obstet. Gynaecol..

[36] Dvash T., Ben-Yosef D., Eiges R. (2006). Human Embryonic Stem Cells as a Powerful Tool for Studying Human Embryogenesis. Pediatr. Res..

[37] Tian W., Zhao M., Li M., Song T., Zhang M., Quan L., Li S., Sun Z.S. (2012). Reversal of Cocaine-Conditioned Place Preference through Methyl Supplementation in Mice: Altering Global DNA Methylation in the Prefrontal Cortex. PLoS ONE.

[38] Novikova S.I., He F., Bai J., Cutrufello N.J., Lidow M.S., Undieh A.S. (2008). Maternal Cocaine Administration in Mice Alters DNA Methylation and Gene Expression in Hippocampal Neurons of Neonatal and Prepubertal Offspring. PLoS ONE.

[39] Watson C.T., Szutorisz H., Garg P., Martin Q., Landry J.A., Sharp A.J., Hurd Y.L. (2015). Genome-Wide DNA Methylation Profiling Reveals Epigenetic Changes in the Rat Nucleus Accumbens Associated With Cross-Generational Effects of Adolescent THC Exposure. Neuropsychopharmacology.

[40] Dholakiya S.L., Aliberti A., Barile F.A. (2016). Morphine Sulfate Concomitantly Decreases Neuronal Differentiation and Opioid Receptor Expression in Mouse Embryonic Stem Cells. Toxicol. Lett..

[41] Hahn J.W., Jagwani S., Kim E., Rendell V.R., He J., Ezerskiy L.A., Wesselschmidt R., Coscia C.J., Belcheva M.M. (2010). Mu and Kappa Opioids Modulate Mouse Embryonic Stem Cell-Derived Neural Progenitor Differentiation via MAP Kinases. J. Neurochem..

[42] Bansal P., Ahern D.T., Kondaveeti Y., Qiu C.W., Pinter S.F. (2021). Contiguous Erosion of the Inactive X in Human Pluripotency Concludes with Global DNA Hypomethylation. Cell Rep..

[43] von Meyenn F., Iurlaro M., Habibi E., Liu N.Q., Salehzadeh-Yazdi A., Santos F., Petrini E., Milagre I., Yu M., Xie Z. (2016). Impairment of DNA Methylation Maintenance Is the Main Cause of Global Demethylation in Naive Embryonic Stem Cells. Mol. Cell.

[44] Leitch H.G., Mcewen K.R., Turp A., Encheva V., Carroll T., Grabole N., Mansfield W., Nashun B., Knezovich J.G., Smith A. (2013). Naive pluripotency is associated with global DNA hypomethylation. Nat. Struct. Mol. Biol..

[45] Montgomery T., Uh K., Lee K. (2024). TET Enzyme Driven Epigenetic Reprogramming in Early Embryos and Its Implication on Long-Term Health. Front. Cell Dev. Biol..

[46] Bartoccetti M., van der Veer B.K., Luo X., Khoueiry R., She P., Bajaj M., Xu J., Janiszewski A., Thienpont B., Pasque V. (2020). Regulatory Dynamics of Tet1 and Oct4 Resolve Stages of Global DNA Demethylation and Transcriptomic Changes in Reprogramming. Cell Rep..

[47] Poole C.J., Lodh A., Choi J.H., van Riggelen J. (2019). MYC deregulates TET1 and TET2 expression to control global DNA (hydroxy)methylation and gene expression to maintain a neoplastic phenotype in T-ALL. Epigenetics Chromatin.

[48] Jimenez-Gonzalez A., García-Concejo A., León-Lobera F., Rodriguez R.E. (2018). Morphine delays neural stem cells differentiation by facilitating Nestin overexpression. Biochim. Biophys. Acta (BBA)-Gen. Subj..

[49] Chen Z.X., Riggs A.D. (2011). DNA Methylation and Demethylation in Mammals. J. Biol. Chem..

[50] He S., Sun H., Lin L., Zhang Y., Chen J., Liang L., Li Y., Zhang M., Yang X., Wang X. (2017). Passive DNA Demethylation Preferentially Up-Regulates Pluripotency-Related Genes and Facilitates the Generation of Induced Pluripotent Stem Cells. J. Biol. Chem..

[51] Zhou W., Wang X., Rosenfeld M.G. (2009). Histone H2A Ubiquitination in Transcriptional Regulation and DNA Damage Repair. Int. J. Biochem. Cell Biol..

[52] Fan X.Y., Shi G., Zhao P. (2019). Reversal of Oxycodone Conditioned Place Preference by Oxytocin: Promoting Global DNA Methylation in the Hippocampus. Neuropharmacology.

[53] Fan X.Y., Shi G., He X.J., Li X.Y., Wan Y.X., Jian L.Y. (2021). Oxytocin Prevents Cue-Induced Reinstatement of Oxycodone Seeking: Involvement of DNA Methylation in the Hippocampus. Addict. Biol..

[54] Sun L., Gu X., Pan Z., Guo X., Liu J., Atianjoh F.E., Wu S., Mo K., Xu B., Liang L. (2019). Contribution of DNMT1 to Neuropathic Pain Genesis Partially through Epigenetically Repressing Kcna2 in Primary Afferent Neurons. J. Neurosci..

[55] Barrot M., Olivier J.D., Perrotti L.I., DiLeone R.J., Berton O., Eisch A.J., Impey S., Storm D.R., Neve R.L., Yin J.C. (2002). CREB activity in the nucleus accumbens shell controls gating of behavioral responses to emotional stimuli. Proc. Natl. Acad. Sci. USA.

[56] Ito S., Shen L., Dai Q., Wu S.C., Collins L.B., Swenberg J.A., He C., Zhang Y. (2011). Tet Proteins Can Convert 5-Methylcytosine to 5-Formylcytosine and 5-Carboxylcytosine. Science.

[57] Iyer L.M., Tahiliani M., Rao A., Aravind L. (2009). Prediction of Novel Families of Enzymes Involved in Oxidative and Other Complex Modifications of Bases in Nucleic Acids. Cell Cycle.

[58] Pastor W.A., Aravind L., Rao A. (2013). TETonic Shift: Biological Roles of TET Proteins in DNA Demethylation and Transcription. Nat. Rev. Mol. Cell Biol..

[59] Tahiliani M., Koh K.P., Shen Y., Pastor W.A., Bandukwala H., Brudno Y., Agarwal S., Iyer L.M., Liu D.R., Aravind L. (2009). Conversion of 5-Methylcytosine to 5-Hydroxymethylcytosine in Mammalian DNA by MLL Partner TET1. Science.

[60] Varambally S., Dhanasekaran S.M., Zhou M., Barrette T.R., Kumar-Sinha C., Sanda M.G., Ghosh D., Pienta K.J., Sewalt R.G.A.B., Otte A.P. (2002). The Polycomb Group Protein EZH2 Is Involved in Progression of Prostate Cancer. Nature.

[61] Karanikolas B.D.W., Figueiredo M.L., Wu L. (2009). Polycomb Group Protein Enhancer of Zeste 2 Is an Oncogene That Promotes the Neoplastic Transformation of a Benign Prostatic Epithelial Cell Line. Mol. Cancer Res..

[62] Li X., Gonzalez M.E., Toy K., Filzen T., Merajver S.D., Kleer C.G. (2009). Targeted Overexpression of EZH2 in the Mammary Gland Disrupts Ductal Morphogenesis and Causes Epithelial Hyperplasia. Am. J. Pathol..

[63] Andrews S. (2010). FastQC: A Quality Control Tool for High Throughput Sequence Data. https://www.bioinformatics.babraham.ac.uk/projects/fastqc/.

[64] Krueger F., James F., Ewels P., Afyounian E., Schuster-Boeckler B. (2019). FelixKrueger/: v0.6.2. https://www.bioinformatics.babraham.ac.uk/projects/trim_galore/.

[65] Granlund T., Stallman R.M. (2018). Cat. https://github.com/Batch-Man/cat.

[66] Langmead B., Salzberg S.L. (2012). Fast gapped-read alignment with Bowtie 2. Nat. Methods.

[67] Langmead B., Wilks C., Antonescu V., Charles R. (2019). Scaling read aligners to hundreds of threads on general-purpose processors. Bioinformatics.

[68] Krueger F., Kreck B., Franke A., Andrews S.R. (2012). DNA methylome analysis using short bisulfite sequencing data. Nat. Methods..

[69] Li H., Handsaker B., Wysoker A., Fennell T., Ruan J., Homer N., Marth G., Abecasis G., Durbin R., 1000 Genome Project Data Processing Subgroup (2009). The Sequence Alignment/Map format and SAMtools. Bioinformatics.

[70] Chen Y., Pal B., Visvader J.E., Smyth G.K. (2017). Differential methylation analysis of reduced representation bisulfite sequencing experiments using edgeR. F1000Research.

[71] Robinson M.D., McCarthy D.J., Smyth G.K. (2010). edgeR: A Bioconductor package for differential expression analysis of digital gene expression data. Bioinformatics.

[72] Akalin A., Kormaksson M., Li S., Garrett-Bakelman F.E., Figueroa M.E., Melnick A., Mason C.E. (2012). methylKit: A comprehensive R package for the analysis of genome-wide DNA methylation profiles. Genome Biol..

[73] Oliveros J.C. Venny. An Interactive Tool for Comparing Lists with Venn’s Diagrams. 2007–2015. https://bioinfogp.cnb.csic.es/tools/venny/index.html.

[74] Ashburner M., Ball C.A., Blake J.A., Botstein D., Butler H., Cherry J.M., Davis A.P., Dolinski K., Dwight S.S., Eppig J.T. (2000). Gene ontology: Tool for the unification of biology. The Gene Ontology Consortium. Nat. Genet..

[75] The Gene Ontology Consortium (2019). The Gene Ontology Resource: 20 years and still GOing strong. Nucleic Acids Res..

[76] Kent W.J., Sugnet C.W., Furey T.S., Roskin K.M., Pringle T.H., Zahler A.M., Haussler D. (2002). The human genome browser at UCSC. Genome Res..

[77] Yohn N.L., Bartolomei M.S., Blendy J.A. (2015). Multigenerational and Transgenerational Inheritance of Drug Exposure: The Effects of Alcohol, Opiates, Cocaine, Marijuana, and Nicotine. Prog. Biophys. Mol. Biol..

[78] Sarkaki A., Assaei R., Motamedi F., Badavi M., Pajouhi N. (2008). Effect of Parental Morphine Addiction on Hippocampal Long-Term Potentiation in Rats Offspring. Behav. Brain Res..

[79] Chorbov V.M., Todorov A.A., Lynskey M.T., Cicero T.J. (2011). Elevated Levels of DNA Methylation at the OPRM1 Promoter in Blood and Sperm from Male Opioid Addicts. J. Opioid Manag..

[80] Saeidinezhad M., Razban V., Safizadeh H., Ezzatabadipour M. (2021). Effects of Maternal Consumption of Morphine on Rat Skeletal System Development. BMC Musculoskelet. Disord..

[81] Sadraie S.H., Kaka G.R., Sahraei H., Dashtnavard H., Bahadoran H., Mofid M., Nasab H.M., Jafari F. (2008). Effects of Maternal Oral Administration of Morphine Sulfate on Developing Rat Fetal Cerebrum: A Morphometrical Evaluation. Brain Res..

