# Supplementary material for: Chronic Morphine Treatment Leads to a Global DNA Hypomethylation via Active and Passive Demethylation Mechanisms in mESCs
Source: Int J Mol Sci. 2025 Jul 22;26(15):7056. doi: 10.3390/ijms26157056 (PMC12345662; doi:10.3390/ijms26157056)
Supplement: Supplementary file 1 [file ijms-26-07056-s001.zip › ijms-3693035-supplementary.pdf]

International Journal of Molecular Sciences

MDPI

SUPPLEMENTARY FILE

Chronic Morphine Treatment Leads to a Global DNA Hypomethylation via Active and Passive Demethylation Mechanisms in mESCs

Manu Araolaza [1,2,†], Iraia Muñoa-Hoyos [1,2,†], Itziar Urizar-Arenaza [1,2], Irune Calzado [1,2] and Nerea Subirán [1,2,*]

[1] Department of Physiology, Faculty of Medicine and Nursery, University of the Basque Country, 48940 Leioa, Spain; manu.araolaza@ehu.eus (M.A.); iraia.munoa@ehu.eus (I.M.H.)
[2] Bizkaia Health Research Institute, 48903 Barakaldo, Spain
* Correspondence: nerea.subiran@ehu.eus; Tel.: +34-946015673
† These authors contributed equally to the work.

Keywords: morphine; development; DNA methylation; mESCs; bioinformatics; passive and active demethylation

ORCID:

Manu Araolaza 0000-0003-1838-4594

Iraia Muñoa-Hoyos: 0000-0002-5107-6991

Itziar Urizar-Arenaza: 0000-0002-6390-7887

Irune Calzado: 0000-0002-1787-6792

Nerea Subiran: 0000-0001-9202-1287

Supplementary Figure S1

A

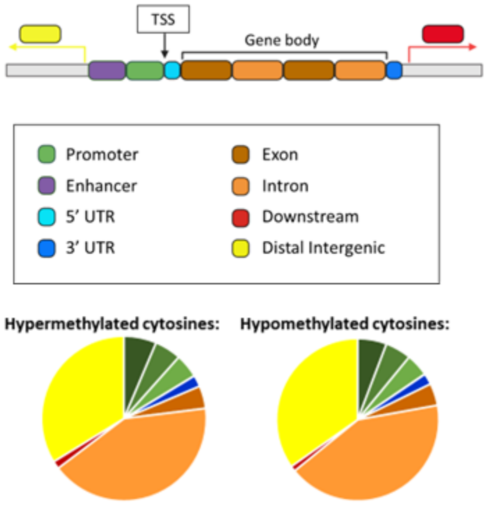

		DMC - Hypermethylations	DMC - Hypomethylations
Promoter – <=1kb		6.4 %	5.8 %
Promoter – 1-2kb		5.2 %	5.2 %
Promoter – 2-3kb		4.7 %	4.5 %
5' UTR		0.0 %	0.1 %
3' UTR		2.2 %	2.2 %
Exon		4.4 %	4.4 %
Intron		41.7 %	42.2 %
Downstream		1.5 %	1.1 %
Distal intergenic		33.8 %	34.8 %

B

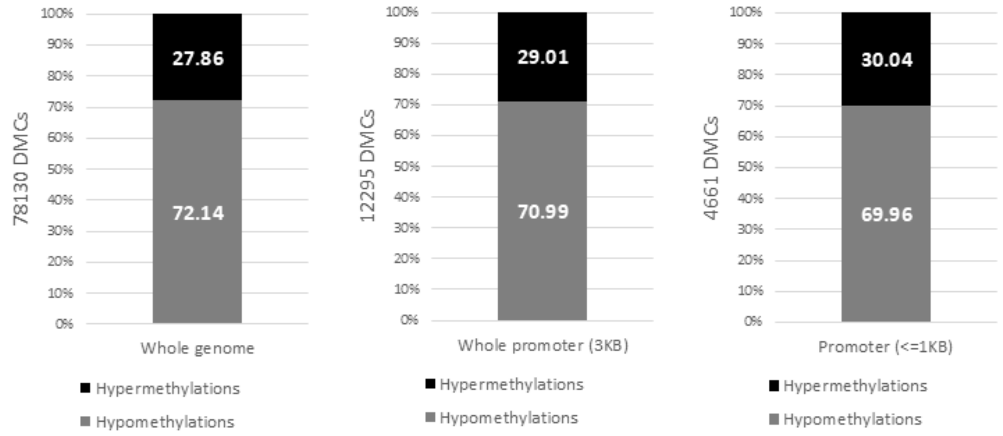

Supplementary fig. S1. Analysis of the distribution of DMCs according genome features. (A) Schematic image of the gene structure, and distribution of DMCs with respect to these gene areas, distinguishing hypermethylated and hypomethylated cytosines; **(B)** Graphs specifying percentage values of hypermethylations and hypomethylations as it approaches the promoter area: DMCs of the whole genome, DMCs that are located less than 3kb from the promoter, and DMCs of the promoter area.

Supplementary Figure S2

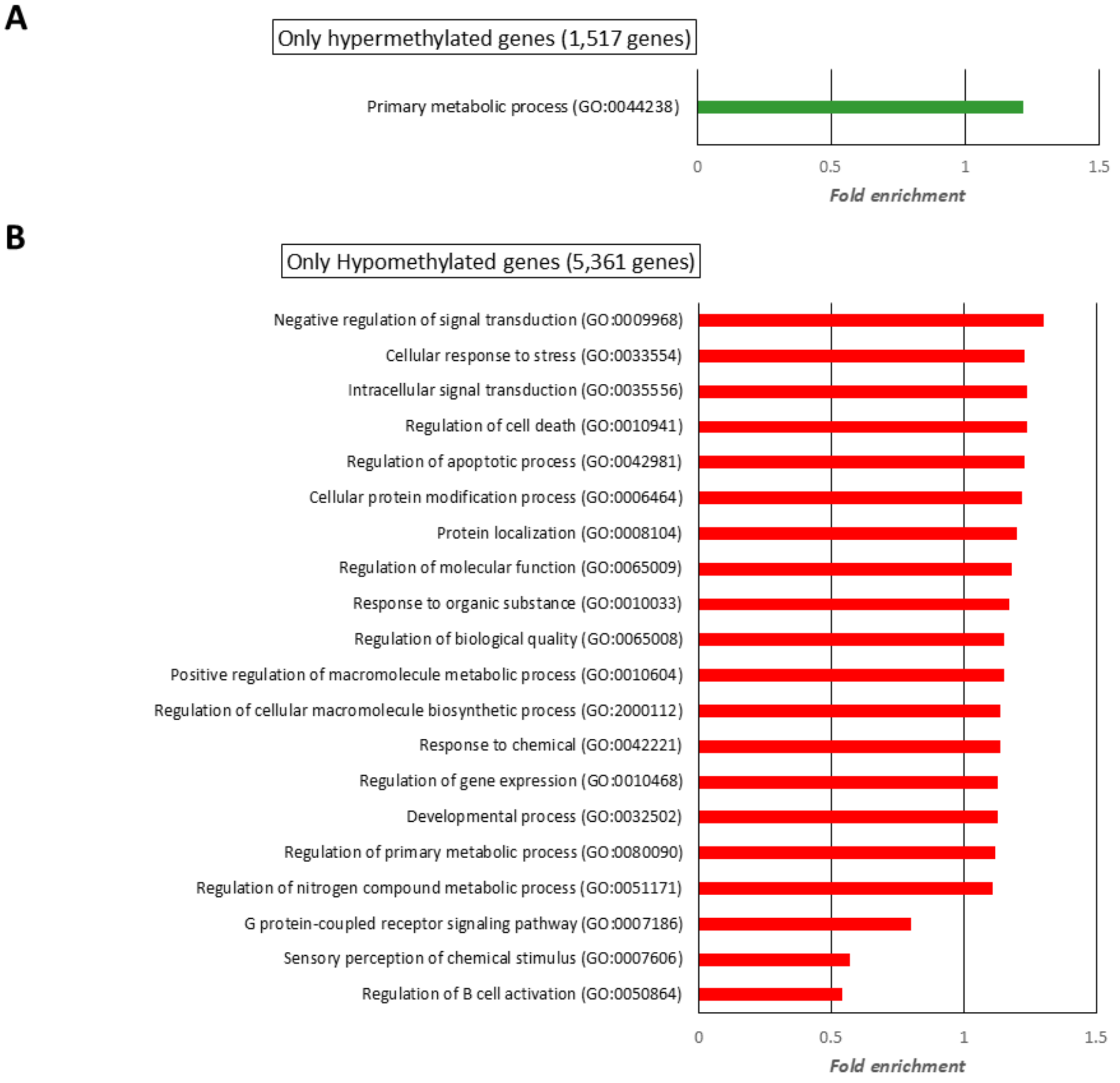

Supplementary fig. S2. Functional enrichment analyses of DMC after chronic morphine treatment. Gene Ontology analysis showing the top biological functions for **(A)** exclusively hypermethylated genes and **(B)** exclusively hypometh-ylated genes. All gene ontology analyses were corrected using Fisher's type test (0,05>p-value).

Supplementary Figure S3

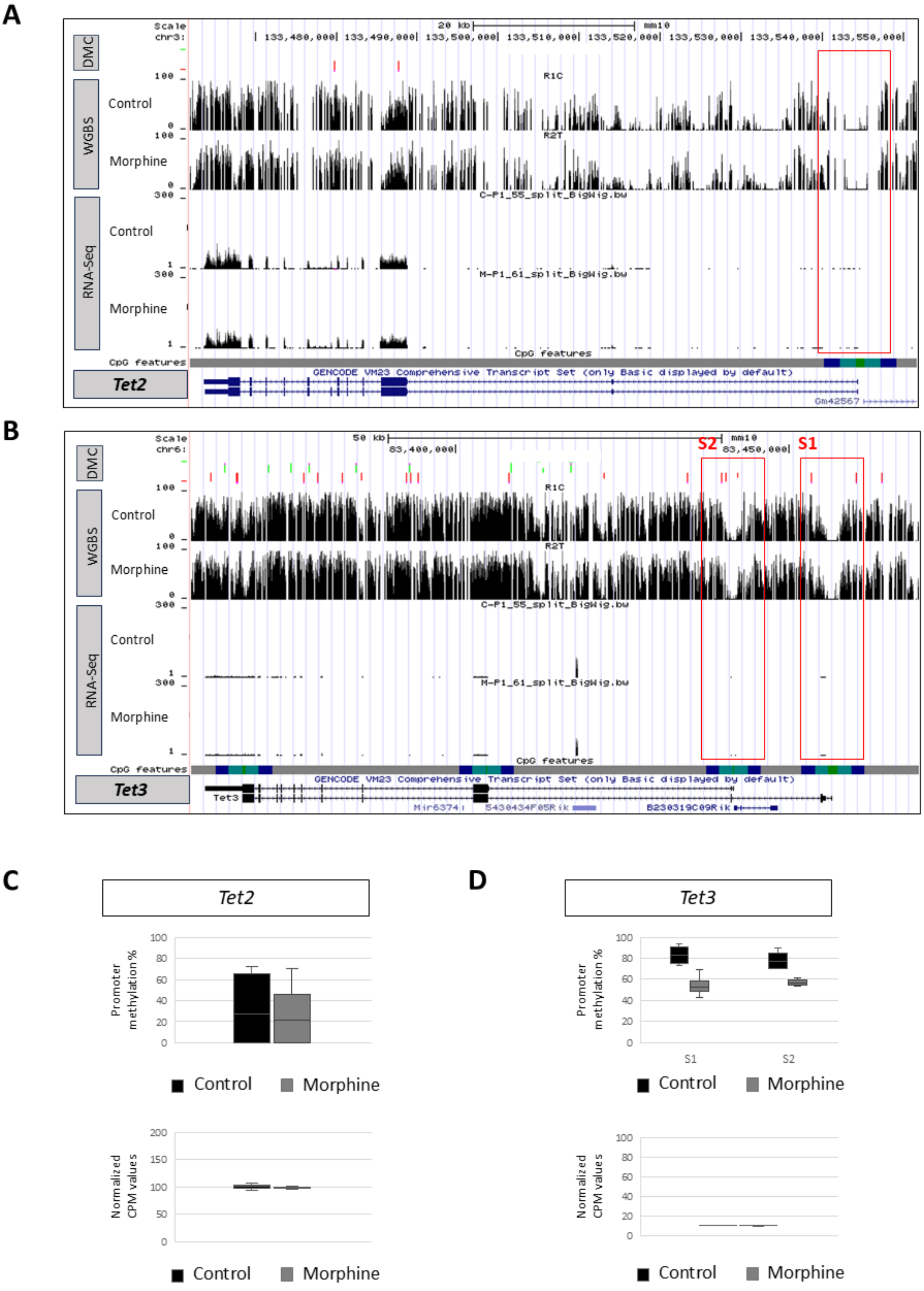

Supplementary fig. S3. Effect of chronic morphine treatment on DNA demethylating proteins. RNA-seq and WGBS track for **(A)** DNA demethylating enzyme Tet2 gene and **(B)** Tet3 gene. CpG features track was composed by CpG islands in green, shores in light blue, shelfs in dark blue and open sea in grey. Red boxes point out the enrichment and gene expression change at promoters. In the DMC row, the light green bars represent hypermethylations, and the red bars, instead, hy-pomethylations. Box and whisker plot showing the percentage of methylation at promoters and CPM values for **(C)** Tet2 gene and **(D)** Tet3 gene after chronic morphine treatment.

Supplementary Figure S4

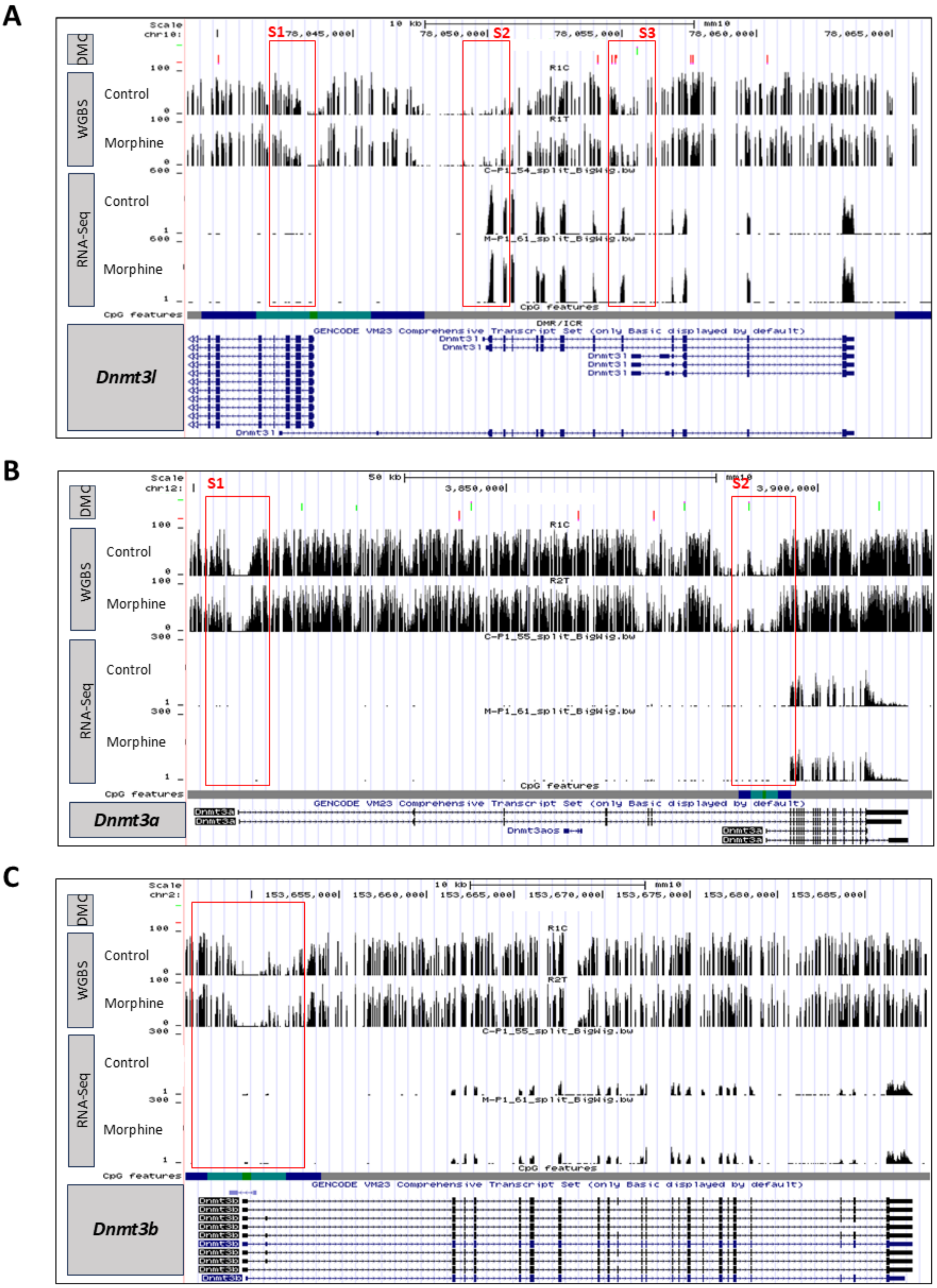

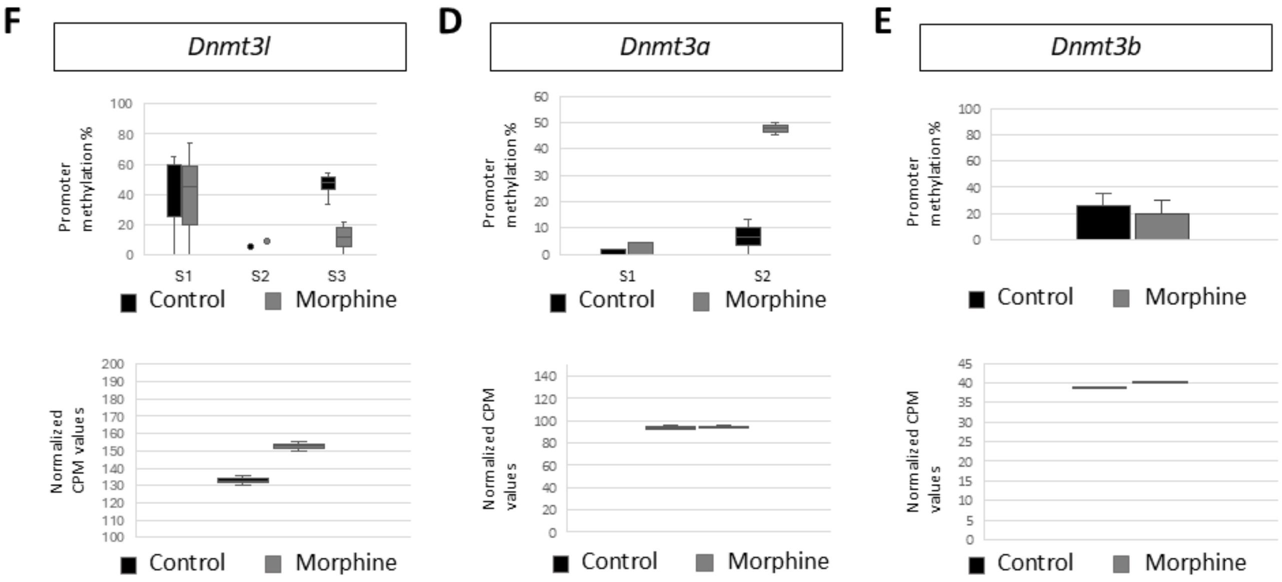

Supplementary fig. S4. Effect of chronic morphine treatment on DNA methylation proteins. RNA-seq and WGBS track for **(A)** DNA methyltransferase Dnmt3l gene, **(B)** Dnmt3a gene, and **(C)** Dnmt3b gene. CpG features track was composed by CpG islands in green, shores in light blue, shelfs in dark blue and open sea in grey. Red boxes point out the enrichment and gene expression change at promoters. In the DMC row, the light green bars represent hypermethylations, and the red bars, instead, hy-pomethylations. Box and whisker plot showing the percentage of methylation at promoters and CPM values for **(D)** Dnmt3l gene, **(E)** Dnmt3a gene and **(F)** Dnmt3b gene after chronic morphine treatment.

Supplementary table S1. Primers used during RT-qPCR assays.

Primers	Sequences
Dnmt1 **(F)**	5'-GCC AGT TGT GTG ACT TGG AA-3'
Dnmt1 **(R)**	5'-GTC TGC CAT TTC TGC TCT CC -3'
Gapdh **(F)**	5'-TAT GAC TCC ACT CAC GGC AAA TT-3'
Gapdh **(R)**	5'-TCG CTC CTG GAA GAT GGT GAT-3'
Pcx **(F)**	5'-CAA CAC CTA CGG CTT CCC TA-3'
Pcx **(R)**	5'-CCA CAA ACA ACG CTC CAT -3'
Tet1 **(F)**	5'-TGC TCC AAA CTA CCC CTT ACA-3'
Tet1 **(R)**	5'-CCC TCT TCA TTT CCA AGT CG-3'
